# Effects of Aquatic Exercise and Floor Curling on Balance Ability and Lower Limb Muscle Strength in Children with Intellectual Disabilities: A Pilot Study in China

**DOI:** 10.3390/children11010085

**Published:** 2024-01-11

**Authors:** Peiting Zhao, Gaohui Zhu, Sha Chen, Yu Pan, Kai Chen, Li Huang, Liya Guo

**Affiliations:** School of Physical Education, Southwest University, Chongqing 400715, China; zptzpt@email.swu.edu.cn (P.Z.); zgh0425@email.swu.edu.cn (G.Z.); cs970904163@email.swu.edu.cn (S.C.); py19980514@email.swu.edu.cn (Y.P.); chenkai@email.swu.edu.cn (K.C.); hl2021@email.swu.edu.cn (L.H.)

**Keywords:** children with intellectual disabilities, balance ability, lower limb muscle strength, aquatic exercise, floor curling

## Abstract

Children with intellectual disabilities often face challenges in balance ability and lower limb muscle strength, which negatively impact their daily lives and motor function. Therefore, it is crucial to enhance the balance ability and lower limb muscle strength of children with intellectual disabilities. This study aimed to investigate the effects of a 12-week aquatic exercise and floor curling intervention on the balance ability and lower limb muscle strength of children with intellectual disabilities. Forty-two participants were randomly assigned to the aquatic exercise group, floor curling group, and control group. The aquatic exercise and floor curling groups received a 12-week intervention, while the control group engaged in supervised free activities. The participants’ balance ability and lower limb muscle strength were assessed using the Berg Balance Scale and a muscle strength testing device before and after the intervention. The results showed significant improvements in balance ability and lower limb muscle strength for both the aquatic exercise group and the floor curling group after the intervention. The aquatic exercise group demonstrated an average improvement of 10.84% in balance ability and an overall average improvement of 16.28% in lower limb muscle strength. The floor curling group showed an average improvement of 9.04% in balance ability and an overall average improvement of 15.67% in lower limb muscle strength. These improvement results were statistically significant (*p* < 0.05) and ranged from medium to large effect sizes (*d* = 0.5~0.8). The findings of this study validate the positive effects of aquatic exercise and floor curling on the balance ability and lower limb muscle strength of children with intellectual disabilities. These interventions can be considered effective approaches for functional rehabilitation in children with intellectual disabilities.

## 1. Introduction

Intellectual disability (ID) is considered to be a neurodevelopmental disorder characterized by a significant decline in intellectual functioning and difficulties with adaptive behaviors, such as communication, self-care, and social skills [1]. It is regarded as one of the most complex disabilities. In addition to the challenges in intellectual functioning and adaptive behaviors, children with ID also face difficulties with physical activities. Research indicates that children with ID may exhibit a higher propensity for motor impairments, such as challenges in balance and coordination, compared to typically developing children [2,3].

Balance ability refers to the capacity of the human body to maintain postural stability in static or dynamic states, which is essential for the normal functioning of daily activities [4]. The maintenance of balance involves the coordinated operation and integration of multiple mechanisms, including the sensory system, central nervous system, and effectors [5]. The sensory input system provides the body with information about its relative position in the surrounding environment. This input information is filtered, integrated, and processed by the central control system, which then issues commands to the effectors. The effectors, such as joint muscles, respond to the received information, thereby controlling or altering body posture [6]. However, cognitive impairments, sensory deficits, and delayed development of joint muscle function in children with ID hinder coordination among the sub-systems responsible for maintaining balance, making them more prone to balance impairments [2,7,8]. Further research has indicated that children and adolescents with ID have lower levels of physical activity, resulting in weaker muscle strength [2,9,10,11], which is closely related to balance ability [12,13,14]. Insufficient balance ability not only increases the risk of falls in these children but also leads to decreased self-management and daily activity of capacity, thereby reducing their motivation to engage in physical activities. Moreover, it can further impede the development of communication and motor skills, limiting their integration into mainstream society and affecting their social adaptation abilities. Therefore, it is crucial to enhance balance ability and closely related lower limb muscle strength in children with ID.

In recent years, aquatic exercise has shown positive effects in the physical functional rehabilitation of patients with neurological disorders [15]. Studies have demonstrated that aquatic exercise, through specific technique-based movements, can lead to significant improvements in balance ability [16,17]. In clinical practice, a range of systematic rehabilitation approaches or specific movement technique exercises are commonly employed to enhance balance ability, such as the Halliwick method and other methods [18], as well as activities like walking and strength training in water [19,20]. These methods and exercise techniques emphasize practicing specific movements and postures in the water to enhance core stability and balance ability and promote functional recovery and independence. Additionally, aquatic exercise utilizes the physical properties of water, such as buoyancy, resistance, and hydrostatic pressure, to provide a resistance-based environment that improves muscle endurance for patients. For individuals with weak muscle strength, water buoyancy can assist in performing active movements, while those with stronger muscle strength can benefit from the resistance of water to increase exercise difficulty and enhance balance ability [18,21]. Based on the aforementioned research and practical evidence, it can be inferred that selecting targeted aquatic exercise as an intervention measure may be beneficial for improving muscle strength and subsequently enhancing balance ability in children with ID.

Meanwhile, we have observed the increasing popularity of floor curling in regions and schools in China, especially in areas where ice rinks are not available [22]. Floor curling has gained significant attention in some special education schools. It is a modified version of the Winter Olympic sport of curling, designed to allow more people to participate. The equipment and rules of floor curling are similar to the Olympic sport, but it is lighter in weight and has three wheels to move on the ground. Floor curling breaks the limitations of traditional ice surfaces, providing opportunities for individuals with disabilities to engage in the sport while promoting inclusivity and diversity. Similar to aquatic exercise, floor curling is a non-contact sport that involves simple mechanical principles and does not make high physical demands, making it easily accessible for individuals with limited sports backgrounds [23]. During the process of floor curling, participants need to coordinate and control their core muscles to maintain balance and stability during dynamic movements and sliding, adapting to the changing demands of balance [24]. Furthermore, the sliding motion in floor curling has the potential to stimulate the sensory system. Therefore, there is reason to believe that utilizing floor curling as an intervention measure may also improve balance ability and lower limb muscle strength in children with ID.

It is worth noting that May et al. conducted a meta-analysis that examined intervention programs targeting balance ability in children with ID. The research findings suggested that interventions with a duration of at least 10 weeks that occur three times per week for 45–60 min per session with moderate exercise intensity yielded optimal results [25]. Chinese scholar Wu Xueping and her team also found that the duration of physical activity recommended by academics is no less than 60 min, and this recommended duration of physical activity can be used as a criterion for evaluating the amount of physical activity in children with ID [26]. Engaging in aquatic exercise and floor curling is advantageous for meeting these requirements, thereby yielding positive effects for children with ID.

In summary, despite the potential benefits of aquatic exercise and floor curling in enhancing balance ability and lower limb muscle strength in children with ID, the current body of research investigating the effects of these interventions on balance ability and lower limb muscle strength in this population remains relatively limited. Therefore, further empirical studies are warranted to validate the effectiveness of aquatic exercise and floor curling as interventions for improving balance ability and lower limb muscle strength in children with ID. These studies would provide additional empirical evidence to support the integration of aquatic exercise and floor curling into functional rehabilitation programs for children with ID.

Our hypotheses are as follows:

**H1.**  *Aquatic exercise intervention improves balance ability and lower limb muscle strength in children with ID.*

**H2.**  *Floor curling intervention improves balance ability and lower limb muscle strength in children with ID.*

## 2. Materials and Methods

### 2.1. Participants

Given the unique characteristics of children with ID and considering the accessibility and willingness of participants, this study employed a convenience sampling approach to recruit parent-supported children with ID from three Special Education Schools and two Intellectual Development Centers in a specific city in Southwest China (*n* = 56). The inclusion criteria for participants were as follows: (a) aged between 9 and 12 years old, (b) possessing basic language comprehension skills to cooperate with the researchers, (c) able to participate in the intervention programs and testing tasks within a designated time with the accompaniment of guardians, and (d) free from any conditions that would restrict participation in aquatic exercise or floor curling, such as heart disease or skin conditions.

To ensure that the sample met the inclusion criteria of this study, a preliminary screening procedure was conducted. Fourteen children did not meet the specified inclusion criteria (*n* = 9) or expressed personal unwillingness to participate (*n* = 5) and were excluded from this study. Subsequently, informed consent was obtained from parents or legal guardians, as well as assent from the participants themselves. Ultimately, a total of 42 children with ID (*n* = 42) were included in the sample, representing the actual participants who completed the intervention and testing protocols.

### 2.2. Procedure

This study employed a randomized controlled trial design, with randomization performed using computer-generated randomization. Participants were randomly assigned to the aquatic exercise group, floor curling group, or control group, with an expected allocation ratio of 1:1:1. Ultimately, 14 participants were assigned to the aquatic exercise group (age = 10.79 ± 1.05, IQ = 39.21 ± 11.98, height = 1.45 ± 0.05 m, weight = 40.51 ± 6.30 kg), 14 participants were assigned to the floor curling group (age = 10.50 ± 1.02, IQ = 38.21 ± 13.73, height = 1.42 ± 0.05 m, weight = 39.81 ± 5.33 kg), and 14 participants were assigned to the control group (age = 10.43 ± 1.10, IQ = 40.14 ± 13.24, height = 1.42 ± 0.07 m, weight = 40.27 ± 5.49 kg).

**Figure 1:**
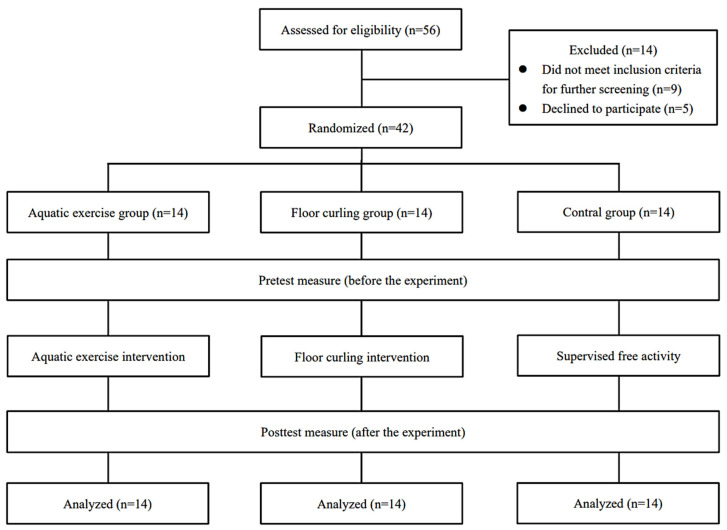
Flowchart of this study. This figure illustrates the grouping and flow of participants in the current study. This study consisted of three groups: the aquatic exercise group, the floor curling group, and the control group, each comprising 14 children with ID who participated in the entire intervention and testing process. No participants withdrew from the intervention due to accidental injuries or other adverse events.

In accordance with the predetermined research design and plan, the exercise intervention used in this study commenced in July 2023 and concluded in October of the same year. The specific procedures are outlined in Figure 1.

### 2.3. Intervention

This study was conducted at a local disability training center with which our research team has established a long-term collaborative relationship. The training center is equipped with sports facilities such as a swimming pool, floor curling rink, tennis courts, and athletics field. It also provides necessary accommodation facilities for children with ID, and it is conveniently located close to the residences of the majority of participants. In addition, this study was based on the recommended moderate physical activity levels in the academic field [25,26], and we set the intervention duration at 12 weeks, with sessions conducted three times per week, each lasting 60 min.

#### 2.3.1. Aquatic Exercise Group

The aquatic exercise group conducts the aquatic exercise intervention in the swimming pool at the training center. The pool has a length of 25 m and a depth of 1.0 m, with the water temperature being maintained at 26–27 °C. Participants are divided into two groups, scheduled for intervention either from 9:00 to 10:00 a.m. or from 10:30 to 11:30 a.m. Four qualified coaches with certification and experience in teaching swimming to children with special needs conducted the interventions in the same environment. Each coach was accompanied by a lifeguard during the interventions, and research team members were present to assist the coaches. The participants’ caregivers were required to be present throughout the intervention.

Considering the lower cognitive abilities and learning skills of children with ID, the aquatic exercise intervention program was a combination of swimming skills and the Halliwick method, following a progressive sequence of movement development, ranging from simple to more complex actions. The aquatic exercise intervention comprises two phases: a 4-week first phase and an 8-week second phase, with three sessions per week. Each session includes 8~10 min of warm-up activities, a 45-min specialized skill practice, and a 5-min relaxation exercises. During the first phase, the focus is on promoting the development of fundamental aquatic movement skills. Thie phase primarily involves practicing basic swimming skills such as breathing, floating, and gliding, as well as the Halliwick ten-point program, which includes mental adjustment, disengagement, transversal rotation control, sagittal rotation control, longitudinal rotation control, combined rotation control, upthrust, balance in stillness, turbulent gliding, simple progression, and basic swimming movement. In the second phase, the exercises introduce freestyle kick techniques from swimming skills. The demands for kick speed and distance gradually increase to enhance muscle strength and body control, ultimately improving balance. An example of a single intervention program for each phase of aquatic exercise is shown in Table 1.

#### 2.3.2. Floor Curling Group

The floor curling group conducts the floor curling intervention at the training center’s floor curling rink. The intervention time and frequency align with those of the aquatic exercise group. Four coaches with qualifications in teaching floor curling, who had received specialized training in special education schools, conducted the interventions in the same environment. Research team members were present to assist the coaches during the interventions, and participants’ caregivers were required to be present throughout the intervention.

The floor curling intervention for children with ID was designed based on their specific developmental characteristics. It followed a progressive sequence of movement development, starting with easier exercises and gradually advancing to more challenging ones. The floor curling intervention consists of two phases: a 4-week first phase and an 8-week second phase, with three sessions per week. Each session includes a 8~10 min warm-up activities, a 45-min specialized skill practice, and a 5-min relaxation exercise. During the first phase, the focus is on promoting the development of fundamental floor curling skills. This phase primarily involves basic floor curling movements and body control exercises, emphasizing individual movement skills or simple combinations such as grip, lunge, and imitation without equipment. In the second phase, the exercises progress to more complex and complete technical practices. Participants are required to further control their body and maintain stable postures during dynamic movements. The repetition and duration of the exercises gradually increase, aiming to enhance muscle strength and body stability, ultimately improving balance. An example of a single intervention program for each phase of floor curling is shown in Table 2.

#### 2.3.3. Control Group

The control group did not participate in organized sports training. Instead, the research team organized three supervised 60-min free activities per week at a nearby sports field. These activities occasionally included physical games.

### 2.4. Outcome and Assessment

#### 2.4.1. Demographics

All participants’ demographic variables were collected using a basic information questionnaire to control for any potential influences of these variables on balance ability and lower limb muscle strength. The research team provided a thorough explanation of this study’s purpose and procedures to the participants’ caregivers, sought input from the children, and obtained parental informed consent before collecting the information. The collected demographic information included participants’ gender, age, height, weight, intelligence quotient level, and other relevant basic details.

Additionally, a Physical Activity Questionnaire for Children (PAQ-C) was administered to the participants’ caregivers to collect information on the participants’ physical activity levels over the past seven days [27]. This was carried out to minimize any potential confounding factors related to balance ability test performance. The PAQ-C has demonstrated good test–retest reliability (R = 0.82) [28].

#### 2.4.2. Balance Ability

In this study, the Berg Balance Test was employed to assess participants’ balance ability. The Berg Balance Scale is known for its accuracy, comprehensiveness, simplicity, and cost-effectiveness [29]. Through 14 tasks, the Berg Balance Scale measures various aspects of participants’ balance, including standing, sitting, transferring, and reaching. Each task is scored on a scale from 0 to 4, with a maximum cumulative score of 56. Higher scores indicate better balance ability. The Berg Balance Scale is considered a reliable tool for assessing individuals with ID, as they can easily understand and follow simple instructions [30]. Additionally, it demonstrates high internal consistency reliability within the ID population [31,32], making it an effective instrument for evaluating the balance abilities of individuals with ID [33].

#### 2.4.3. Lower Limb Muscle Strength

In this study, the participants’ lower limb muscle strength was assessed using a handheld dynamometer (HHD) known as the Hoggan^®^ MicroFET3. The HHD is portable, easy to use, and highly sensitive, making it suitable for assessing lower limb muscle strength in various populations, including children with cerebral palsy [34], traumatic brain injury [35], neuromuscular disorders [36], and ID (ICC = 0.81–0.96) [37]. The HHD has demonstrated good test–retest reliability in assessing lower limb muscle strength in these populations. Additionally, our assessors received professional training, and there were no changes in assessors between pre- and post-testing.

Based on the mechanisms involved in maintaining balance, this study measured the strength of participants’ hip extensors, hip flexors, knee extensors, knee flexors, toe extensors, and ankle dorsiflexors. The measurement results were recorded in kilograms (KG) to quantify the maximum strength of these specific muscle groups. The measurement procedures are as follows:

(1) Hip extensor muscles: The participant assumes a prone position with the knees flexed at a 90° angle. The testing device is positioned near the popliteal fossa of the posterior thigh, and force is generated to extend the hip joint.
(2) Hip flexor muscles: The participant sits with both feet suspended and the lower legs naturally hanging. The testing device is placed near the anterior thigh muscles close to the patella, and force is applied to flex the hip joint.
(3) Knee extensor muscles: The participant sits with the popliteal region near the edge, and both feet are suspended with the lower legs naturally hanging. The testing device is positioned in front of the tibia, with the concave surface aligned with the line connecting the medial and lateral malleoli. Force is exerted to extend the knee joint.
(4) Knee flexor muscles: The participant assumes a prone position, with the knee joint flexed at a 90° angle. The testing device is positioned at the Achilles tendon, aligning the concave surface with the line connecting the medial and lateral malleoli. Force is applied to flex the knee joint.
(5) Toe extensor muscles: The participant lies supine with the knee joint extended and the foot in a natural neutral position. The testing device is positioned at the metatarsals of the plantar surface, and force is applied to perform toe extension.
(6) Ankle dorsiflexor muscles: The participant lies supine with the knee joint extended and the foot in a natural neutral position. The testing device is positioned at the dorsal surface of the metatarsals, and force is applied to perform ankle dorsiflexion.

### 2.5. Statistical Analyses

The normality and homogeneity of variance were confirmed using the Shapiro–Wilk test and Levene’s test, respectively. Continuous variables following a normal distribution were presented as means (*M*) ± standard deviations (*SD*).

Firstly, a one-way analysis of variance (ANOVA) was performed to examine the measurement differences among groups at baseline (pre-test) and post-test. The effect size of ANOVA was calculated using Eta squared (*η*^2^) and based on the criteria proposed by J. Cohen, and the effect sizes were classified as follows: small (*η*^2^ = 0.01), medium (*η*^2^ = 0.06), and large (*η*^2^ = 0.14) [38]. In the event of significant differences revealed by the one-way ANOVA, post hoc multiple comparisons using the LSD method were conducted to determine specific group differences. Subsequently, paired-samples *t*-tests were employed to detect within-group differences before and after the intervention. The effect size for the *t*-test was computed using Cohen’s d, and according to J. Cohen’s criteria, the effect sizes were categorized as small (*d* = 0.2), medium (*d* = 0.5), and large (*d* = 0.8) [39].

All statistical tests were two-tailed, with a significance level set at *p* < 0.05, indicating statistical significance. The statistical analysis in this study was conducted using IBM SPSS 26.0 (SPSS Inc., Chicago, IL, USA).

## 3. Results

### 3.1. Baseline Characteristics

As shown in Table 3, there were no significant differences between the three groups of participants in terms of age, body mass index (BMI), physical activity, intelligence quotient (IQ), or other variables at baseline (pre-test). Additionally, no statistically significant differences were found among the three groups in the Berg Balance Test and lower limb muscle strength measures.

### 3.2. Between-Group Differences

Table 4 presents the results of the Berg Balance Test and lower limb muscle strength tests conducted after intervention for the three groups of participants. The aquatic exercise group, floor curling group, and control group exhibited significant between-group differences in both the Berg Balance Test and lower limb muscle strength tests (*p* < 0.05). Post hoc multiple comparisons using the LSD method were performed to further validate the differences between the groups.

**Figure 2:**
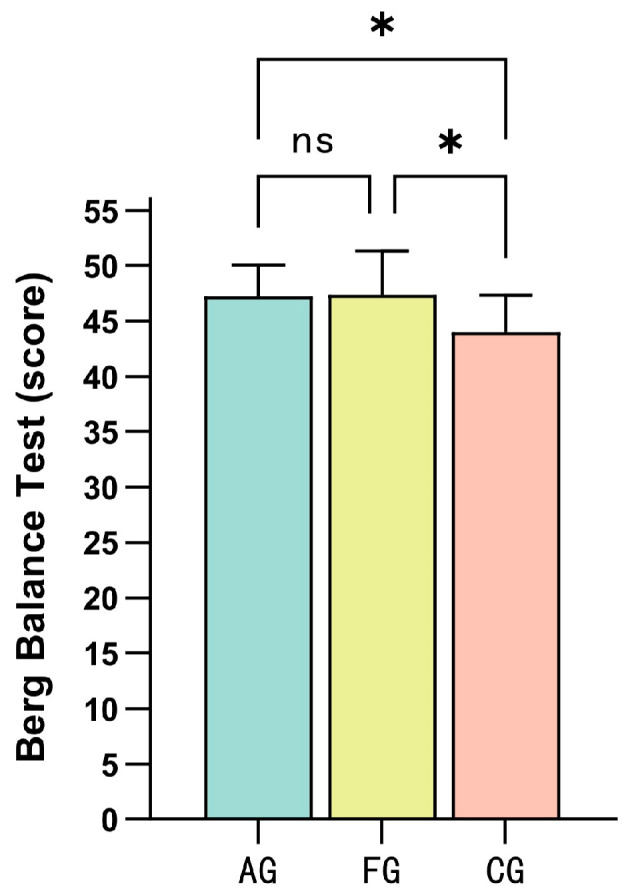
Post hoc LSD multiple comparison results of one-way ANOVA for the Berg Balance Test. Note: AG = aquatic exercise group; FG = floor curling group; CG = control group; ns, *p* > 0.05; *, *p* < 0.05.

Figure 2 displays the results of the LSD post hoc multiple comparisons for the Berg Balance Test between the groups. The between-group comparison between the aquatic exercise group and the floor curling group revealed no significant difference after the intervention (*p* = 0.913, 95%CI = −2.756 to 2.470). However, a significant difference was observed between the aquatic exercise group and the control group (*p* = 0.017, 95%CI = 0.601 to 5.827), as well as between the floor curling group and the control group (*p* = 0.013, 95%CI = 0.744 to 5.970). These findings suggest that there were significant improvements in balance ability for the aquatic exercise group and the floor curling group compared to the control group after a 12-week intervention period.

**Figure 3:**
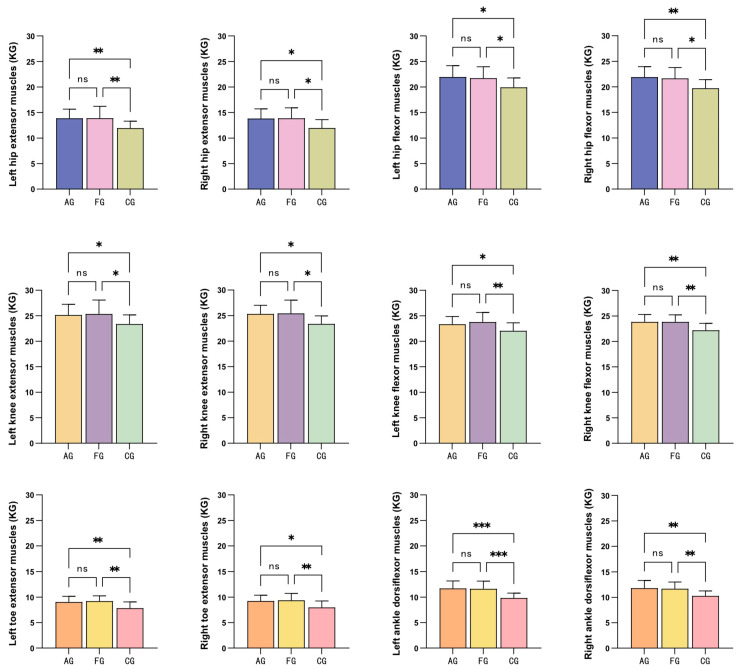
Post hoc LSD multiple comparison results of one-way ANOVA for the Berg Balance Test. Note: AG = aquatic exercise group; FG = floor curling group; CG = control group; ns, *p* > 0.05; *, *p* < 0.05; **, *p* < 0.01; ***, *p* < 0.001.

Figure 3 presents the results of the LSD post hoc multiple comparisons for the lower limb muscle strength between the groups. The results of the lower limb muscle strength tests indicated that there were no significant differences between the aquatic exercise group and the floor curling group after the intervention (left hip extensor muscles: *p* = 0.984, 95%CI = −1.449 to 1.421; right hip extensor muscles: *p* = 0.936, 95%CI = −1.488 to 1.373; left hip flexor muscles: *p* = 0.783, 95%CI = −1.393 to 1.837; right hip flexor muscles: *p* = 0.731, 95%CI = −1.244 to 1.759; left knee extensor muscles: *p* = 0.802, 95%CI = −1.932 to 1.503; right knee extensor muscles: *p* = 0.882, 95%CI = −1.656 to 1.427; left knee flexor muscles: *p* = 0.494, 95%CI = −1.712 to 0.840; right knee flexor muscles: *p* = 0.979, 95%CI = −1.094 to 1.065; left toe extensor muscles: *p* = 0.667, 95%CI = −1.053 to 0.682; right toe extensor muscles: *p* = 0.788, 95%CI = −1.091 to 0.834; left ankle dorsiflexor muscles: *p* = 0.888, 95%CI = −0.950 to 1.093; right ankle dorsiflexor muscles: *p* = 0.805, 95%CI = −0.865 to 1.108).

However, significant differences were observed between the aquatic exercise group and the control group (left hip extensor muscles: *p* = 0.009, 95%CI = −3.378 to −0.508; right hip extensor muscles: *p* = 0.013, 95%CI = −3.267 to −0.405; left hip flexor muscles: *p* = 0.016, 95%CI = −3.630 to −0.399; right hip flexor muscles: *p* = 0.006, 95%CI = −3.673 to −0.670; left knee extensor muscles: *p* = 0.044, 95%CI = −3.482 to −0.047; right knee extensor muscles: *p* = 0.015, 95%CI = −3.477 to −0.394; left knee flexor muscles: *p* = 0.047, 95%CI = −2.569 to −0.017; right knee flexor muscles: *p* = 0.004, 95%CI = −2.730 to −0.057; left toe extensor muscles: *p* = 0.008, 95%CI = −2.075 to −0.340; right toe extensor muscles: *p* = 0.011, 95%CI = −2.241 to −0.316; left ankle dorsiflexor muscles: *p* = 0.001, 95%CI = −2.893 to −0.850; right ankle dorsiflexor muscles: *p* = 0.003, 95%CI = −2.508 to −0.535), as well as between the floor curling group and the control group (left hip extensor muscles: *p* = 0.009, 95%CI = −3.392 to −0.522; right hip extensor muscles: *p* = 0.011, 95%CI = −3.324 to −0.462; left hip flexor muscles: *p* = 0.031, 95%CI = −3.408 to −0.178; right hip flexor muscles: *p* = 0.014, 95%CI = −3.416 to −0.413; left knee extensor muscles: *p* = 0.025, 95%CI = −3.696 to −0.261; right knee extensor muscles: *p* = 0.010, 95%CI = −3.592 to −0.508; left knee flexor muscles: *p* = 0.009, 95%CI = −3.005 to −0.453; right knee flexor muscles: *p* = 0.003, 95%CI = −2.744 to −0.585; left toe extensor muscles: *p* = 0.002, 95%CI = −2.260 to −0.525; right toe extensor muscles: *p* = 0.005, 95%CI = −2.370 to −0.445; left ankle dorsiflexor muscles: *p* = 0.001, 95%CI = −2.822 to −0.778; right ankle dorsiflexor muscles: *p* = 0.007, 95%CI = −2.387 to −0.414). These findings suggest that a 12-week intervention of aquatic exercise and floor curling resulted in significant changes in lower limb muscle strength compared to the control group.

### 3.3. Within-Group Differences

Paired sample *t*-test results revealed statistically significant differences (*p* < 0.01) in the Berg Balance Test and lower limb muscle strength measures before and after the interventions for both the aquatic exercise group and the floor curling group, indicating the significant impact of the interventions in these two groups. However, no differences were observed in the control group before and after the intervention (*p* > 0.05).

Table 5 presents the mean changes and estimated effect sizes for each group in the pre- and post-tests. Regarding the Berg Balance Test, the aquatic exercise group exhibited large effect sizes (*d* > 0.8), while for the lower limb muscle strength measures, the aquatic exercise group showed moderate (*d* > 0.5) to large effect sizes (*d* > 0.8). Similar effects were observed in the floor curling group, suggesting a stable and similar effect size for the intervention. In contrast, the effect sizes in the control group were below the minimum threshold of meaningful effect (*d* < 0.2). These findings indicate that aquatic exercise and floor curling have positive impacts on the balance ability and lower limb muscle strength of children with ID.

## 4. Discussion

The aim of this study was to investigate the effects of aquatic exercise intervention and floor curling intervention on the balance ability and lower limb muscle strength of children with ID. The results revealed significant positive changes in the Berg Balance Test scores and lower limb muscle strength measures after a 12-week intervention period of both aquatic exercise and floor curling. These changes were accompanied by moderate to large effect sizes. In contrast, the control group did not show significant improvements in balance ability and lower limb muscle strength before and after the intervention. The findings of this study further support the two research hypotheses proposed earlier in this paper.

### 4.1. Effects of Aquatic Exercise on Balance Ability and Lower Limb Muscle Strength in Children with Intellectual Disabilities

Previous research has demonstrated the beneficial effects of aquatic exercise on balance ability in individuals with neurological disorders. Kurt et al. conducted a 5-week aquatic exercise intervention for individuals with Parkinson’s disease and observed significant improvements in balance ability following the intervention [40]. Similarly, for children and adolescents with neurological disorders, aquatic exercise interventions have proven beneficial. Ansari et al. revealed significant enhancements in balance abilities among 8–14-year-old children with Autism Spectrum Disorder (ASD) following a 10-week aquatic exercise intervention [41]. The results of this study showed that after 12 weeks of aquatic exercise intervention, balance ability and lower limb muscle strength improved in children with ID.

The intervention program implemented in this study focused on practicing fundamental aquatic movement skills and body control and the development of lower limb strength. During exercises such as floating, gliding, rotating, and leg movements, children with ID actively engaged in dynamic movements and performed static exercises involving selective muscle contractions and stability training to better control their body positions in the water. These exercises aimed to promote the mutual development of static and dynamic balance, continuously stimulate their sensory perceptual and body control abilities, and strengthen the “stimulus-response” loop. Moreover, this underscores the essential role of researchers in closely manipulating aquatic exercise interventions. By designing and guiding aquatic exercise intervention programs appropriate to the developmental level of children with ID, researchers can create a wide range of sensory-stimulating environments. These environments offer various sensory inputs and movement challenges, thereby providing opportunities for children to engage in meaningful sensory experiences and motor skill development.

This study observed significant improvements in Berg Balance Test scores, hip and knee flexor–extensor muscle groups, and foot muscle groups in children with ID. During aquatic exercise, the lower limb muscles of children with ID are continuously active and bear loads. Specifically, maintaining balance and stability in the water during basic swimming skills and body control exercises is achieved through the coordination of core muscles and lower limb muscles [42]. Therefore, this continuous stability training is beneficial for improving lower limb muscle strength and balance ability. In further freestyle kick exercise, greater muscle strength is required to generate propulsion and maintain stability in the water. This sustained muscle activity and load are advantageous for stimulating the growth and development of lower limb muscles, further enhancing lower limb muscle strength.

It is noteworthy that in this study, although the Halliwick ten-point program was only integrated into the exercise content of the first phase of aquatic exercise intervention, the exercises within this program may have facilitated improvements in balance ability and lower limb muscle strength in children with ID. The Halliwick method emphasizes training in posture control, with a core principle of establishing stable posture control before engaging in independent movements, as trunk stability is crucial for efficient limb propulsion during swimming [43]. Consequently, exercises such as transversal rotation control require participants to strengthen their muscle control to counteract the forces and inertia generated by rotational movements. Simultaneously, they need to adjust their body’s central position in a timely manner to maintain balance and coordinate limb movements to support balance control during rotation. In longitudinal rotation control, participants also need to dynamically adjust their body posture to maintain balance, involving adjustments in the position of the head, trunk, and legs, as well as employing stretching, flexing, and twisting movements to sustain balance. Moreover, visual and sensory feedback play a vital role in perceiving the rotational state and making corresponding adjustments to maintain balance [44].

In addition to the aforementioned aquatic exercise content, the unique environment provided by water may play a distinct role in improving balance ability and lower limb muscle strength in children with ID. Firstly, the static hydrostatic pressure exerted by water can stimulate proprioceptors, enhancing individuals’ kinesthetic awareness and perception of body movement and position [45,46]. Secondly, during aquatic exercise, gentle vestibular stimulation can be provided through the movement of water and pressure on the body. The vestibular system responsible for spatial orientation and balance can be trained and improved through this stimulation [47]. Thirdly, water resistance activates and strengthens muscles. The resistance provided by water represents the weight to be overcome during freestyle kick exercise. Continuous resistance from water is crucial for increasing muscle strength while minimizing excessive joint loading, reducing the impacts on joints and bones and making the training safer and more comfortable [48,49]. Additionally, aquatic exercise involves complex movement patterns and coordination demands. Through repeated practice and increased difficulty, the nervous system adapts and improves control over these movements. This neural adaptation promotes the transmission of neural impulses, thereby enhancing coordination and reaction speed in lower limb muscles and improving balance ability [50].

### 4.2. Effects of Floor Curling on Balance Ability and Lower Limb Muscle Strength in Children with Intellectual Disabilities

Based on previous research on curling, it is known that this sport requires excellent balance and core stability [51]. Moreover, the throwing technique in curling places high demands on hip joint flexibility and strength [52]. Previous studies have also demonstrated that elite curlers exhibit superior balance ability [53]. While the movement patterns and techniques in curling and floor curling are similar, research on the effects of floor curling interventions on the balance ability and lower limb muscle strength of individuals with neurological disorders remains relatively limited. Therefore, this study employed the more inclusive approach of floor curling as an intervention for children with ID. The findings of this study revealed significant improvements in Berg Balance Test performance, hip and knee flexor–extensor muscle groups, and foot muscle groups following a 12-week floor curling intervention.

The floor curling intervention program utilized in this study emphasized the practice of fundamental techniques, body stability, and lower limb muscle strength. This activity involves squatting, pushing the curling stone, and coordinating the muscle activity of the hip, knee, and foot to maintain vertical throwing around the support base, thus establishing and sustaining proper body balance [24]. To maintain a stable throwing posture and proper body alignment, the intervention program incorporates targeted exercises for foundational body stability, such as toe balance and straight-line walking, which are highly beneficial for children with ID who are new to floor curling. These exercises enhance joint flexibility and flexibility and establish necessary lower limb muscle strength and body stability [54,55]. During the specialized technique practice for throwing the curling stone, children with ID must stand stably on the ground and maintain body stability while pushing the stone, placing higher demands on their balance ability. Therefore, they require stronger lower limb muscle strength to maintain stability during the stone’s propulsion. By gradually increasing the difficulty of the exercises and repeatedly practicing these movement skills, lower limb muscles gradually gain strength and endurance, thereby improving balance ability.

It is worth mentioning that floor curling is a relatively safe land-based sport [56], but the unilateral technique actions and relatively fixed body postures may increase the risk of joint strain [52]. Therefore, the floor curling exercises used in this study ensured equal practice frequency for both sides of the body and included auxiliary exercises for body stability. Such exercise design helps to reduce the potential risks of imbalance and excessive unilateral loading on the joints, promoting the balanced development of balance ability and muscle strength coordination in children with ID.

Furthermore, the motor development patterns of children with ID are similar to those of typically developing children [57]. The improvement in balance ability among children with ID through floor curling may be attributed to the promotion of eye–foot coordination, perceptual abilities, and lower limb muscle strength [58]. Floor curling requires children with ID to control the speed and direction of the stone through pushing, which demands fine coordination and perceptual abilities, including hand–eye coordination and coordination control between different body parts. Through floor curling training, their neural systems’ control over movements can be enhanced, and muscle coordination and reaction speed can be improved. Through contact with the stone and the floor, children with ID can experience different sensory feedback related to sliding on the ground and pushing the stone. Such sensory stimulation can promote the development of perceptual abilities and enhance participants’ perception and understanding of body position, movement, and the environment. Additionally, based on previous research on curling training, targeted curling skill training may reduce spinal reflex activity, thereby decreasing muscle activation around joints (such as the knees and ankles) and fundamentally preventing joint oscillation, which is unnecessary and uncontrollable for maintaining balance [59,60,61]. This, in turn, may contribute to improved balance ability.

### 4.3. Limitations and Recommendations for Future Research

While the results of this study contribute to understanding the potential benefits of aquatic exercise and floor curling interventions for children with ID, it is essential to acknowledge certain limitations in the research.

Firstly, the sample size of this study was relatively small, which is attributed to the uniqueness and accessibility of children with ID. Within the study’s targeted region and accessible formal recruitment channels, there was a higher proportion of male children with ID. Among the children who met the criteria for further screening and whose parents expressed willingness to participate in the study, only male children remained eligible. Therefore, this study only included a sample of male children with ID. Although a smaller sample size facilitates researchers’ control over the quality of experiments [62], future research should aim to incorporate a more diverse population of children with ID, including both males and females, to gain a more comprehensive understanding of their responses and effects in motor interventions.

Secondly, despite the exclusion of participants with conditions that make exercise unsuitable, such as heart diseases, future research should consider monitoring physiological indicators such as heart rate to understand the physiological responses of children with ID to exercise. Such monitoring can provide more accurate data and assist in developing personalized physical activity recommendations suitable for children with ID [63].

Furthermore, it is important to acknowledge that while both aquatic exercise and floor curling can improve balance ability and lower limb muscle strength in children with ID, they differ in terms of environmental characteristics, neuroadaptation, sensory stimulation, and muscular activity and strength requirements. Each intervention elicits distinct stimuli that activate different neural pathways involved in maintaining balance, making direct comparisons difficult [64]. Future research should further design comparative experiments to better understand the differential effects of different exercise interventions and provide more guidance for selecting appropriate interventions for individuals.

Lastly, due to limitations in research time and resources, this study did not conduct multiple measurements of key indicators during the intervention process. This limitation may have increased the impact of data fluctuations on the results and led to us potentially overlooking the influence of time factors. In future research, these aspects should be appropriately considered to track changes in key indicators during the intervention process.

## 5. Conclusions

This study investigated the effects of aquatic exercise and floor curling on balance ability and lower limb muscle strength in children with ID. The findings revealed the following conclusions: (1) A 12-week intervention of aquatic exercise and floor curling significantly improved the balance ability and lower limb muscle strength of children with ID. (2) Both aquatic exercise and floor curling interventions emphasized specialized basic technique exercises, body control exercises, and movements conducive to the development of lower limb muscle strength. Furthermore, the technical movements practiced were consistent with the dynamic characteristics of the assessments. Consequently, post-test data indicated significant improvements in both the balance ability and lower limb muscle strength of the participants. (3) Supervised free activities did not have a notable effect in terms of improving balance ability and lower limb muscle strength in children with ID.

In summary, aquatic exercises and floor curling show potential for the functional rehabilitation of children with ID. Therefore, they can be further considered for inclusion in rehabilitation treatment programs for children with ID.

## Figures and Tables

**Table 1:** Activity examples for the aquatic exercise intervention plan. Phase 1 is a combination of basic swimming skills and the Halliwick ten-point program, while Phase 2 is the freestyle kick exercise in swimming skills.

Phase	Exercise Program	Example Activity	Duration
1	Preparation activities		8~10 min
	Warm-up	Jogging, Free-Standing Exercises.	
	Fundamental aquatic skills practice		5 min
	Deep breathing in water	Execute a series of 15 deep breaths to enhance respiratory control.	
	Floating practice	Attain an unassisted prone float for a duration of 10 s.	
	Halliwick ten-point program		40 min
	Transversal rotation control	Ability to control any rotation around the front cross axis.	
	Sagittal rotation control	Ability to control any rotation around the sagittal axis (anterior/posterior).	
	Longitudinal rotation control	Ability to control any rotation around the sagittal (longitudinal) axis.	
	Relaxation exercises		5 min
	Free activity	Drilling into the swimming circle and floating freely in the water.	
	Deep breathing in water	Continuously take 15 deep breaths.	
2	Preparation activities		8~10 min
	Warm-up	Jogging, Free-Standing Exercises.	
	Fundamental aquatic skills practice		5 min
	Deep breathing in water	Execute a series of 30 deep breaths to enhance respiratory control.	
	Floating practice	Attain an unassisted prone float for a duration of 15 s.	
	Freestyle kick		40 min
	Poolside leg striking exercises	6 sets of 60 s.	
	Freestyle kick	20 × 25 m freestyle kick.	
	Relaxation exercises		5 min
	Free activity	Drilling into the swimming circle and floating freely in the water.	
	Deep breathing in water	Continuously take 30 deep breaths.	

**Table 2:** Activity examples for the floor curling intervention plan. Phase 1 is the practice of basic floor curling skills and body control exercises, while Phase 2 is the practice of more complex and complete floor curling skills.

Phase	Exercise Program	Example Activity	Duration
1	Preparation activities		8~10 min
	Warm-up	Jogging, Free-Standing Exercises.	
	Body control exercises		15 min
	Walk on tiptoe	Tiptoe walk, 4 sets of 7.5 m.	
	Lunge exercise	Squat in a lunge position and hold for 10 s, practicing 4 sets of 10 s on each side of the leg.	
	Floor curling basic moves exercise		
	Curling grip exercise	Hook the curling handle with the fingers together with force, hold the curling handle with the palm of the hand empty and the tiger’s mouth in the direction of the opposite shoulder position.	5 min
	Unarmed imitation curling practice	Once the lunge is complete, the opposite hand simulates pushing the curling motion, practicing 4 sets of 6 reps on each side.	25 min
	Relaxation stretching		5 min
	Stretching exercise	Stretching exercise.	
2	Preparation activities		8~10 min
	Warm-up	Jogging, Free-Standing Exercises.	
	Body control exercises		15 min
	Walk on tiptoe	Tiptoe walk, 4 sets of 15 m.	
	One-legged stand	One foot off the ground, hold for 1 min, but you can drop it halfway through, 2 sets of 1 min each on the left and right sides.	
	Squat exercise	Squat and stand up, 3 sets of 10 reps.	
	Curling moves practice		30 min
	Pushing movements	Exercises using cardboard for pushing movements, 5 sets of 10 reps.	
	Pushing movements	Use a floor curler for pushing movements, 5 sets of 10 reps.	
	Floor curling games	Floor curling passes in pairs.	
	Relaxation		5 min
	Relaxation stretching	Stretching exercise.	

**Table 3:** Descriptive statistic (*M* ± *SD*) and one-way ANOVA of baseline demographic characteristics, Berg Balance Test, and lower limb muscle strength tests for each group. No significant differences were found between the three groups.

	Aquatic Exercise Group (*n* = 14)	Floor Curling Group (*n* = 14)	Control Group (*n* = 14)	*F*	*p*	*η* ^2^
	*M* ± *SD*	*M* ± *SD*	*M* ± *SD*			
Age (years)	10.79 ± 1.05	10.50 ± 1.02	10.43 ± 1.09	0.450	0.641	0.023
BMI (kg/m^2^)	19.25 ± 1.90	19.69 ± 1.91	19.94 ± 1.57	0.541	0.586	0.027
PAQ-C (score)	1.81 ± 0.35	1.78 ± 0.20	1.74 ± 0.26	0.238	0.790	0.012
IQ (score)	39.21 ± 11.98	38.21 ± 13.73	40.14 ± 13.24	0.077	0.926	0.004
Berg (score)	42.64 ± 2.92	43.50 ± 4.07	43.71 ± 3.02	0.394	0.677	0.020
Lower limb muscle strength (KG)						
Left hip extensor muscles	10.88 ± 1.65	10.88 ± 2.62	11.74 ± 1.96	0.766	0.472	0.038
Right hip extensor muscles	11.13 ± 1.79	10.75 ± 2.24	11.86 ± 1.82	1.161	0.324	0.056
Left hip flexor muscles	18.89 ± 2.28	19.40 ± 2.50	19.76 ± 2.05	0.506	0.607	0.025
Right hip flexor muscles	18.84 ± 2.37	19.16 ± 2.77	19.78 ± 1.66	0.593	0.557	0.030
Left knee extensor muscles	23.57 ± 2.07	23.74 ± 2.72	22.95 ± 2.20	0.442	0.646	0.022
Right knee extensor muscles	23.81 ± 2.13	23.63 ± 2.71	23.02 ± 1.95	0.461	0.634	0.023
Left knee flexor muscles	21.46 ± 1.32	21.82 ± 1.77	21.96 ± 1.66	0.367	0.695	0.018
Right knee flexor muscles	21.56 ± 1.38	21.79 ± 1.44	22.51 ± 1.84	1.410	0.256	0.067
Left toe extensor muscles	7.54 ± 1.12	7.86 ± 1.22	7.63 ± 1.42	0.242	0.787	0.012
Right toe extensor muscles	7.95 ± 1.27	7.98 ± 1.43	8.09 ± 1.35	0.044	0.957	0.002
Left ankle dorsiflexor muscles	9.84 ± 1.26	10.00 ± 1.40	9.69 ± 0.94	0.234	0.792	0.012
Right ankle dorsiflexor muscles	9.97 ± 1.44	10.41 ± 1.10	10.37 ± 1.01	0.573	0.569	0.029

Note: PAQ-C = Physical Activity Questionnaire for Children; IQ = intelligence quotient, based on the ICD-10 (International Classification of Diseases, 10th edition) criteria for intellectual disabilities.

**Table 4:** Descriptive statistics (*M* ± *SD*) and one-way ANOVA of the Berg Balance Test and lower limb muscle strength tests after the intervention for each group. Significant differences between the three groups.

	Aquatic Exercise Group (*n* = 14)	Floor Curling Group (*n* = 14)	Control Group (*n* = 14)	*F*	*p*	*η* ^2^
	*M* ± *SD*	*M* ± *SD*	*M* ± *SD*			
Berg (score)	47.21 ± 2.83	47.36 ± 3.99	44.00 ± 3.33	4.319	0.020	0.181
Lower limb muscle strength (KG)						
Left hip extensor muscles	13.88 ± 1.79	13.89 ± 2.33	11.94 ± 1.39	5.038	0.011	0.205
Right hip extensor muscles	13.81 ± 1.92	13.87 ± 2.04	11.98 ± 1.63	4.633	0.016	0.192
Left hip flexor muscles	21.95 ± 2.23	21.73 ± 2.23	19.94 ± 1.85	3.827	0.030	0.164
Right hip flexor muscles	21.91 ± 2.06	21.65 ± 2.12	19.74 ± 1.68	5.110	0.011	0.208
Left knee extensor muscles	25.14 ± 2.10	25.36 ± 2.73	23.38 ± 1.81	3.270	0.049	0.144
Right knee extensor muscles	25.31 ± 1.71	25.43 ± 2.61	23.38 ± 1.56	4.570	0.016	0.190
Left knee flexor muscles	23.34 ± 1.53	23.78 ± 1.86	22.05 ± 1.59	4.062	0.025	0.172
Right knee flexor muscles	23.82 ± 1.47	23.84 ± 1.40	22.17 ± 1.37	6.426	0.004	0.248
Left toe extensor muscles	9.02 ± 1.12	9.21 ± 1.03	7.81 ± 1.24	6.220	0.005	0.242
Right toe extensor muscles	9.24 ± 1.13	9.37 ± 1.37	7.96 ± 1.26	5.346	0.009	0.215
Left ankle dorsiflexor muscles	11.70 ± 1.47	11.63 ± 1.51	9.83 ± 0.95	8.817	0.001	0.311
Right ankle dorsiflexor muscles	11.81 ± 1.51	11.69 ± 1.33	10.29 ± 0.98	6.011	0.005	0.236

**Table 5:** Within-group changes and effect sizes for pre- and post-test data of each group.

	Aquatic Exercise Group (*n* = 14)		Floor Curling Group (*n* = 14)		Control Group (*n* = 14)	
	Change *M* ± *SD*	*d*	Change *M* ± *SD*	*d*	Change *M* ± *SD*	*d*
Berg (total score)	4.57 ± 1.55	1.56	3.86 ± 1.83	0.95	0.29 ± 1.49	0.09
Lower limb muscle strength (KG)						
Left hip extensor muscles	3.00 ± 1.34	1.82	3.01 ± 0.89	1.15	0.20 ± 0.99	0.10
Right hip extensor muscles	2.69 ± 1.19	1.50	3.13 ± 0.87	1.40	0.12 ± 0.44	0.07
Left hip flexor muscles	3.06 ± 1.04	1.34	2.33 ± 1.37	0.93	0.18 ± 0.75	0.09
Right hip flexor muscles	3.06 ± 1.18	1.29	2.49 ± 1.11	0.90	−0.04 ± 0.57	−0.03
Left knee extensor muscles	1.57 ± 0.52	0.76	1.61 ± 0.71	0.59	0.43 ± 0.67	0.19
Right knee extensor muscles	1.50 ± 0.93	0.71	1.80 ± 0.63	0.66	0.36 ± 0.86	0.18
Left knee flexor muscles	1.89 ± 1.52	1.43	1.96 ± 0.51	1.10	0.09 ± 1.33	0.06
Right knee flexor muscles	2.26 ± 0.55	1.64	2.05 ± 0.10	1.42	−0.34 ± 0.74	−0.19
Left toe extensor muscles	1.49 ± 0.35	1.33	1.35 ± 0.62	1.10	0.19 ± 1.11	0.13
Right toe extensor muscles	1.29 ± 0.70	1.02	1.39 ± 0.42	0.97	−0.13 ± 1.19	−0.10
Left ankle dorsiflexor muscles	1.86 ± 0.34	1.48	1.63 ± 0.80	1.16	0.143 ± 0.60	0.15
Right ankle dorsiflexor muscles	1.84 ± 0.56	1.28	1.28 ± 0.50	1.17	−0.09 ± 0.81	−0.08

Note: The effect size for the *t*-test was computed using Cohen’s *d*, and according to J. Cohen’s criteria, the effect sizes were categorized as small (*d* = 0.2), medium (*d* = 0.5), and large (*d* = 0.8) [39].

## Data Availability

The original datasets generated supporting the conclusion of this study are available on request to the corresponding author, without undue reservation. The data are not publicly available due to its association with the content of the author’s unfinished graduate thesis.
